# Determinants of low birth weight in neonates born in three hospitals in Brong Ahafo region, Ghana, 2016- an unmatched case-control study

**DOI:** 10.1186/s12884-019-2315-6

**Published:** 2019-05-16

**Authors:** Zakariah Adam, Donne Kofi Ameme, Priscillia Nortey, Edwin Andrew Afari, Ernest Kenu

**Affiliations:** 1 Ghana Field Epidemiology and Laboratory Trainning Programme, Department of Epidemiology and Disease Control, University of Ghana School of Public Health, Legon, Ghana; 2 Ghana Health Service, Bolgatanga Regional Health Directorate, Bolgatanga, Ghana

**Keywords:** Low birth weight, Brong Ahafo region, Case control study

## Abstract

**Background:**

Each year, about 20 million Low Birth Weight (LBW) babies are born with very high proportion (96.5%) occuring in developing countries. In the last decade, the incidence of LBW in Ghana has not declined. Brong Ahafo Region of Ghana recorded a LBW prevalence of 11% which was higher than the the national average of 10%. This study identifed determinants of LBW delivery in the Brong Ahafo Region.

**Methods:**

We conducted a 1:2 unmatched case control study among mothers with singleton deliveries in 3 major health facilities in the Brong Ahafo Region. A case was defined as a mother who delivered a baby weighing less than 2500g in any of the three selected health facilities between 1st December, 2015 and 30th April, 2016. A control was defined as a mother who within 24 h of delivery by a case, delivered a baby weighing at least 2500g and not exceeding 3400g in the same health facility. Deliveries that met the inclusion criteria for cases were selected and two controls were randomly selected from the pool of deliveries that meet criteria for controls within 24 h of delivery of a case. A total of 120 cases and 240 control were recruited for the study. We computed odds ratios at 95% confidence level to determine the associations between low birth weight and the dependent factors.

**Results:**

After controlling for confounders such as planned pregnancy, mode of delivery, parity and previous LBW in stepwise backward logistic regression, first trimester hemoglobin &lt; 11 g/dl (aOR 3.14; 95%CI: 1.50–6.58), delivery at 32-36 weeks gestation (aOR 13.70; 95%CI: 4.64–40.45), delivery below 32 weeks gestation (aOR 58.5; 95%CI 6.7–513.9), secondary education of mothers (aOR 4.19; 95%CI 1.45–12.07), living with extended family (aOR 2.43; 95%CI 1.15–5.10, living alone during pregnancy (aOR 3.9; 95%CI: 1.3–11.7), and not taking iron supplements during pregnancy (aOR 3.2; 95%CI: 1.1–9.5) were found to be significantly associated with LBW.

**Conclusion:**

Determinants of LBW were: preterm delivery, mothers with secondary education, living alone during pregnancy, not taking daily required iron supplementation and mothers with first trimester hemoglobin below 11 g/dl. Education during antenatal sessions should be tailored to address the identified risk factors in the mother and child health care services.

## Background

Low birth weight (LBW) remains a major issue of public health concern globally with a disproportionate burden on low and middle income countries. LBW contributes significantly to morbidity, mortality and disability in neonatal, infancy, and childhood periods. It also has long term effects on health outcomes in adult life.

Low birth weight contributes 60 to 80% of all neonatal deaths [1]. Therefore, reducing the incident rates of LBW, is essential to reducing child mortality [2]. Progress made in reducing neonatal mortality has been slower in Sub-Saharan Africa compared to any other region in the world [3]. The incidence of LBWs in Ghana for 2014 was 160 per 1000 births. Ghana has not recorded any reduction in LBW in the last decade [4]. In one of the three major tertiary hospitals in Ghana, 16.5% of the 11,647 babies born were low birth weight in the year 2013 [5]. Also about 46% of all LBW babies in Ghana die in the first 28 days of life [6]. In the Brong Ahafo region the incidence of LBW was 11.0% in 2014, this is higher than the national incidence of 10%. In addition to the LBW of the region being consistently higher than the national average since 2010, it recorded 397 neonatal deaths in 2014 [7]. Together, the hospitals selected for the study recorded 1149 (11.4%) LBW babies out of the 10,075 live births in the region for 2015 [7]. Care for low birth weight babies comes with substantial costs to the health system and imposes a significant burden on the society as a whole [8]. The weight of a newborn is an important predictor of the infant’s growth and survival [9, 10]. LBW babies from birth are disadvantaged and their survival is very poor. LBW babies have been known to have a higher risk of death compared to infants of normal weight, [11]. A Canadaian study also confirmed that LBW was a significant factor associated with neonatal death [12]. There are risk factors that increase a pregnant woman’s chance of having a low birth weight baby. These include: smoking, alcohol consumption, underweight, being younger than 15 years, or older than 35 years. Also the social and economic factors, such as low income, low educational level, stress, domestic violence or other abuse, and being an unmarried woman may increase the risk of a low birth weight delivery. [13–16]. LBW is usually an indicator of chronic maternal malnutrition, maternal illness and poor prenatal care, hence, a good indicator of the socioeconomic status of inhabitants [17, 18].

Low birth weight infants are predisposed to both short and long term health problems. Some short term conditions include, respiratory distress syndrome, infections, enterocolitis, hydrocephalus, and mental retardation [19]. Some of the long term conditions are: coronary heart disease, non-insulin dependent diabetes, childhood hypertension, behavioral disorders, impaired cognitive function, and psychological disorders [20–23]. In the Brong Ahafo Region of Ghana, little is known about the factors that predispose the pregnant woman to delivery of a LBW baby. Therefore, identifying these factors will significantly contribute to ongoing efforts to address LBW. A reduction in the incidence of LBW will reduce the cost of care of the LBW to the healthcare system and eventually reduce neonatal and child mortality.

## Methods

### Study design

We carried out a 1:2 an unmatched case control study in three major hospitals in Brong Ahafo Region, Ghana from 1st December 2015 to 30th April 2016. These hospitals recorded the largest number of deliveries per year in the region and serve as the major referral centres in the region.

### Study site

The study was conducted in the Brong Ahafo Regional Hospital, Sunyani Municipal Hospital and the Holy Family Hopsital.

Brong Ahafo Regional Hospital is the main referral centre in the region for patients requiring secondary healthcare services. In 2015, data from DHIMS revealed that the hospital recorded a total of 3261 live births with 12.6% (410/3261) being low birth weight. The hospital has one gynecological theatre, one labour ward, three post-delivery wards and one neonatal unit.

Holy family hospital serves as another referral centre for medical conditions including obstetric and gynecological conditions. It is located in Techiman, which is considered the busiest trading centre of the region. Data from the DHIMS for 2015 showed that the hospital recorded a total of 5152 live births with 12.4% (641/5152) low birth weight babies. The hospital one labour ward, a post-delivery ward and one neonatal unit.

The Sunyani Municipal Hospital is the third referral hospital and it serves the Sunyani Municipality. It recorded a total of 1662 live births with 5.9% (98/1662) low birth weights for 2015 (DHIMS 2015). It has one theatre, one labour ward and one post-delivery ward. All the data collected from this hospital were in the records of the post-delivery ward.

### Case selection

#### Case

A case was defined as a mother who delivered a baby weighing less than 2500g in any of the three selected health facilities between 1st December, 2015 and 30th April, 2016.

#### Control

A control was defined as a mother who within 24 h of delivery by a case, delivered a baby weighing at least 2500g and not exceeding 3400g in the same health facility.

#### Inclusion criteria

Mothers with singleton deliveries of babies whose weight is 3400 g or less. Mothers who consent to participate in the study.

#### Exclusion criteria

Babies with congenital abnormalities or still births. Mothers who are critically ill.

### Sample size calculation

A sample size calculation formula for unmatched case control study was used with the following parameters: power of the study was 80%, Z_β_ = 0.84 and at 0.05 significance level, Z_α_ = 1.96. The proportion exposed in the control group used was 33%, thus the exposure was nulliparity in a study carried out in The Gambia (Jammeh et al., 2011).

Minimal detectable odds ratio that was used was 2. Based on this a total of 360 case control respondent pairs, that is a 1:2 unmatched case control pairs was arrived at. An additional 15% was added to adjust for missing data.

### Data collection technique and tools

A data collection questionnaire was designed to collect data from mothers who delivered and met the criteria to be included in the study. The questionnaire obtained both primary and secondary data. We obtained the secondary data by reviewing the antenatal and postnatal health records of the mother. The questionnaire was pretested in a health facility with similar characteristics as the study sites. The questionnaire was revised to improve clarity of some of the questions.

The required data from mothers with low birth weight babies were collected within 24 h upon delivery. Data was collected each time a low birth weight baby was delivered until the required sample size was obtained. The data collection officer visited the post-delivery, labour, and neonatal wards three times each day (morning, afternoon and evening) to identify study participants. Also, the staff on duty at the post delivery, labour, and neonatal units alerted the data collection officer each time a delivery meeting the case definition and inclusion criteria occurred. Data was collected by administering the structured questionnaire to the mother and also recording information from the mothers’ antenatal records book and the maternity ward records. Data were collected concurrently in all the three health facilities until the total required sample size was obtained.

Two controls were selected on the same day of delivery of a case by a simple random method. Where more than two controls were delivered within 24 h after delivery of the case, the controls are assigned numbers which were entered into a random digit selection software to select the controls randomly. This was done concurrently in all the three health until the required sample size was reached.

Consenting mothers were taken to an office within the ward for the questionnaire to be administered to ensure confidentiality.

The data collected included:

Socio demographic information: age, occupation of mother, educational status, income status, baby’s sex, marital status, social support status, height, weight, residence, and planning of pregnancy.

Obstetric data included: gestation at booking, gestation at delivery, mode of delivery, family planning methods used, previous abortions, previous delivery of a LBW baby, parity, number of Antenatal Care (ANC) visits.

Medical status information: any chronic medical condition, illness during pregnancy, hospital admissions during pregnancy, intake of required daily dose of iron supplementation, appetite during pregnancy, use of herbal medications during pregnancy and alcohol intake during pregnancy.

Data collection was carried out with the help of two data collection officers. The officers were health professionals trained in the area of maternal and child health. They were selected from the facilities used for the study. They were trained a week prior to commencement of the data collection. They were then introduced to the heads of departments where recruitment was done.

Data was cross-checked for errors and entered using EpiInfo 7 software. Data was saved in password protected files and no one had access to it except for cross-referencing. Filled questionnaires were kept in locked cabinets. Participants were identified by codes.

### Data processing and analysis

Data were analyzed using STATA software Version 13. Continuous variables were summarized into means and proportions, whiles categorical variables were summarized into frequencies.

Bivariate analysis was done between birthweight and each of the independent variables to determine the associations using the chi-square test of proportions. The odds ratios and confidence intervals were reported using 95% level of significance. All variables in the bivariate analysis with a *p*-value of less than 0.05 was considered for multiple logistic regression analysis. The backward stepwise logistic regression model was used to test for the determinant predictors for LBW. The level of significance for regression analysis was set at 95%.

### Ethical consideration

We obtained ethical approval from the Ghana Health Service Ethics Review Committee (GHS/ERS 071015). Also, permission was obtained from the Brong Ahafo Regional Health Directorate and the Heads of the Sunyani Regional Hospital, Sunyani Municipal Hospital and Holy Family Hospital to access the participants and their records. The study was explained to participants and their concerns addressed. A written informed consent was obtained from all participants. Each consenting participant signed or thumb printed on the consent form before the questionnaire was administered. For participant who were under 18, consent was sought form their guardians, and the participant provided a written assent to take part in the study. Both the mother or guardian and participant signed a consent form before the interviews were conducted.

## Results

### Socio demographic characteristics of study participants

A total of 120 cases and 240 controls were studied in the three health facilities. The mean age of the mothers was 28.2 ± 5.9 yeras for cases and 29.0 ± 5.9 years for controls). About 10.1% (12/120) of the cases and 11.8% (28/240) of ths had no formal education. Majority of the mothers; 61.1% (73/120) cases and 73.5% (176/240) of controls lived in urban areas (Table 1).

**Table 1:** Socio-demographic characteristics of study participants, Brong Ahafo Region, 2016

Variable	No. of Cases (%) (*n* = 120)	No. of Control (%) (*n* = 240)
Age group of mother (years)		
19 and below	10 (8.3)	18 (7.5)
20–29	51 (42.5)	103 (42.9)
30+	59 (49.2)	119 (49.6)
Marital status of mother		
Married	83 (69.2)	186 (78.5)
Not married	37 (30.8)	51 (21.5)
Mothers occupation		
Farming	17 (14.3)	28 (11.8)
Trader/artisan	63 (52.9)	136 (57.1)
Government/formal job	16 (13.4)	33 (13.9)
Unemployed	23 (19.3)	41 (17.2)
Residence of mother		
Urban	69 (61.1)	172 (73.5)
Rural	44 (38.9)	62 (26.5)

### Anthropometric characteristics of study participants

Most of the cases, 97.2%(103/120), and controls, 97.8%(221/240) were at least 1.46m tall. Majority of the cases, 56.9%(58/120), and controls, 63.3%(138/240) had normal BMI. The associations between the anthropometric measurements and low birth weight (LBW) are summarized in Table 2.

**Table 2:** Bivariate analysis of anthropometric characteristics of study participants, Brong Ahafo Region, 2016

Variables	Cases(*N* = 120) N (%)	Controls (*N* = 240) N (%)	OR	95% CI	*p*-value
Mothers height(m)					
1.46 m or above (normal)	103 (97.2)	221 (97.8)	1		ref
Below 1.46 m(short)	3 (2.8)	5 (2.2)	1.29	0.20–6.75	0.50 ^˄^
Mothers weight					
45 kg and above	107 (97.3)	219 (96.5)	1		ref
Below 45 kg	3 (2.7)	8 (3.5)	0.77	0.13–3.27	0.50 ^˄^
BMI of mother (kg/m^2^)					
18.5–24.9 (normal)	58 (56.9)	138 (63.3)	1		ref
Below 18.5 (underweight)	3 (2.9)	13 (6.0)	0.55	0.15–2.01	0.36
Above 24.9(obese)	41 (40.2)	67 (30.7)	1.46	0.89–2.40	0.14

˄-fischer exact *p*-value

### Obstetric characteristics of study participants

Majority of the mothers had a first trimester ANC attendance [64.8% (68/120) among cases and 69.1% (161/240) among controls]. Most of them had attended ANC at least four times during the pregnancy period (82.2% of cases and 95.8% of controls). Booking in the 3rd trimester (OR;5.2, 95%CI:1.70–15.98):, fewer than three ANC visits (OR; 4.94, 95%CI: 2.12–11.12), unplanned pregnancy (OR:1.6, 95%CI: 1.00–2.53), delivery by caesarian section (OR:1.64, 95%CI:1.00–2.68), primiparity (OR: 2.66, 95%CI: 1.09–6.48), and previous delivery of a LBW/premature baby (OR: 2.6, 95%CI: 0.95–7.31) were significantly associated with delivery of a LBW baby (Table 3).

**Table 3:** Obstetric characteristics of study participants, Brong Ahafo Region, 2016

Variables	Cases (*N* = 120) N (%)	Controls (*N* = 240) N (%)	OR	95% CI	*P*-value
Gestation at booking					
1st trimester	68 (64.8)	161 (69.1)	1		ref
2nd trimester	26 (24.8)	67 (28.8)	0.92	0.54–1.57	0.76
3rd trimester	11 (10.5)	5 (2.1)	5.21	1.70–15.98	< 0.001
ANC visits					
4 and above	97 (82.2)	228 (95.8)	1		Ref
0–3	21 (17.8)	10 (4.2)	4.94	2.12–11.12	< 0.001
Planned pregnancy					
Yes	64 (53.8)	155 (65.1)	1		ref
No	55 (46.2)	83 (34.9)	1.6	1.00–2.53	0.038
Mode of delivery					
Spontaneous	73 (61.3)	172 (72.3)	1		Ref
Caesarian section	46 (38.7)	66 (27.7)	1.64	1.00–2.68	0.036
Parity					
Nulliparity	44 (37.3)	62 (26.1)	2.66	1.09–6.48	0.024
Primiparity	21 (17.80	55 (23.1)	1.43	0.56–3.65	0.45
Multiparty(2–3)	45 (38.1)	91 (38.2)	1.85	0.78–4.41	0.16
Grand multiparity(4+)	8 (6.8)	30 (12.6)	1		ref
Family planning method					
No	84 (70.0)	185 (77.1)	1		Ref
Yes	36 (30.0)	55 (22.9)	1.45	0.86–2.44	0.14
Previous abortion					
No	96 (80.0)	192 (80.0)	1		ref
Yes	24 (20.0)	48 (20.0)	1.01	0.55–1.79	0.99
Previous LBW/premature Delivery					
No	109 (90.8)	232 (96.3)	1		ref
Yes	11 (9.2)	9 (3.7)	2.6	0.95–7.31	0.034

### Maternal health characteristics of study participants

The hemoglobin level of 66.6% (240/360) of the mothers with low birth weight babies was below 11g/dl, whiles 47.9% (172/360) of the mothers with normal weight babies had theirs below 11g/dl. First trimester hemoglobin (OR: 2.18, 95%CI:1.34–3.55), hospital admissions during pregnancy (OR:2.65, 95%CI: 1.59–4.92), no iron supplementation intake (OR:2.83, 95%CI:137–5.85) and intake of herbal preparations (OR: 3.02, 95%CI:1.57–5.84) were significantly associated with delivery of a LBW in the bivariate analysis (Table 4).

**Table 4:** Bivariate analysis of the maternal health characteristics of study participants, Brong Ahafo Region, 2016

Variables	Cases(*N* = 120) N (%)	Controls(*N* = 240) N (%)	OR	95% CI	*p*-value
First trimester hemoglobin					
11 g/dl and above	39 (33.3)	124 (52.1)	1	ref	
Below 11 g/dl	78 (66.6)	114 (47.9)	2.18	1.34–3.55	< 0.001
Hospital admissions in pregnancy					
No	72 (60.0)	191 (79.9)	1	ref	
Yes	48 (40)	48 (20.1)	2.65	1.59–4.42	< 0.001
Iron supplementation intake					
Always	69 (57.5)	175 (73.2)	1	ref	
Not regularly	32 (26.7)	47 (19.7)	1.73	1.01–2.94	0.042
Never	19 (15.8)	17 (7.1)	2.83	1.37–5.85	< 0.001
Poor appetite					
No	49 (41.2)	122 (52.6)	1	ref	
Yes	70 (58.8)	110 (47.4)	1.58	0.99–2.54	0.043
Poor appetite					
No	49 (41.2)	122 (52.6)	1		ref
Yes	70 (58.8)	110 (47.4)	1.58	0.99–2.54	0.043
Intake of Herbal medications					
No	92 (76.7)	218 (90.8)	1		ref
Yes	28 (23.3)	22 (9.2)	3.02	1.57–5.84	< 0.001

### Determinants of LBW among study participants

On multiple logistic regression, first trimester hemoglobin below 11g/dl, delivery at 32-36 weeks gestational age, delivery before 32 weeks gestational age, and secondary education of mother were significantly associated with LBW (Table 5).

**Table 5:** Determinants of Low Birth Weight among study participants, Brong Ahafo Region, 2016

Characteristics	Cases *N* = 120 (%)	Controls *N* = 240 (%)	cOR (95%CI)	aOR (95%C)
Gestation at delivery				
Term delivery(37–40 weeks)	76 (63.3)	228 (95.8)	1	1
Preterm delivery(32-36 weeks)	32 (26.4)	9 (3.8)	10.5* (4.46–24.68)	13.70* (4.64–40.45)
Severe preterm delivery (< 32 weeks)	12 (10.3)	1 (0.4)	61.89* (6.84–560.33)	58.50* (6.66–513.87)
Educational level of mother				
Post-secondary/tertiary	16 (13.4)	43 (18.1)	1	1
Primary/JHS	35 (29.4)	101 (42.0)	0.94 (0.47–1.88)	1.15 (0.39–3.40)
Secondary	57 (47.1)	68 (28.2)	2.25* (1.13–4.47)	4.19* (1.45–12.07)
No formal education	12 (10.1)	28 (11.8)	1.16 (0.47–2.81)	1.88 (0.46–7.63)
Supplementary iron intake				
Regular intake	69 (57.5)	175 (73.2)	1	1
Irregular intake	32 (26.7)	47 (19.7)	1.73* (1.01–2.94)	2.19 (0.99–4.84)
No intake	19 (15.8)	17 (7.1)	2.83* (1.37–5.85)	3.19* (0.99–4.84)
Who mother lived with during pregnancy				
Partner	55 (46.2)	157 (65.4)	1	1
Extended family	41 (34.5)	63 (26.2)	1.86* (1.12–3.09)	2.43* (1.15–5.10)
Lived alone	23 (19.3)	20 (8.4)	3.24* (1.62–6.48)	3.95* (1.33–11.74)
1st trimester hemoglobin				
11 g/dl and above	39 (33.3)	125 (52.1)	1	1
Below 11 g/dl	78 (66.6)	115 (47.9)	2.18* (1.34–3.55)	3.14* (1.50–6.58)
Hospital admissions during pregnancy				
No	72 (60.0)	192 (79.9)	1	1
Yes	48 (40)	48 (20.1)	2.65* (1.59–4.42)	2.08 (1.00–4.30)

* *p*-value for the odds ratio is less than 0.05

*OR* Odds Ratio, *aOR* Adjusted Odds Ratio, *CI* Confidence Interval

## Discussion

The study was conducted to identify determinants of LBW delivery in the three selected health facilities in the Brong Ahafo region of Ghana.

Women younger than 19 years had an increased odds of delivering a low birth weight baby. This is because, adolescent mothers may not have a fully developed reproductive system. A study by Guimares and colleagues has shown that adolescent (< 20 years of age) mothers have poorer socioeconomic and reproductive conditions as compared with older age groups and this increases the risk of having a LBW baby [24]. With respect to educational level, mothers with secondary education had a four-fold increased odds of delivering a LBW baby compared to mothers with tertiary education. In keeping with findings from our study, a meta-analysis by Silverstrin and his team showed that having a higher education had a protective effect, whereas having a medium education had no protective effects [25] .

Social support which is a measure of support from family, the baby’s father, and general functional support is a necessary factor for good pregnancy outcomes [26]. Lack of social support has been associated with increased risk of LBW [27]. In our study the social support system referred to who the mother lived with during the pregnancy. The study shows a four fold increased odds of having a LBW delivery for a mother who lived alone, compared with one who lived with a partner or the unborn baby’s father during the pregnancy. A study by Feldman showed that social support was linked with infant birth weight through processes of fetal growth [26]. This suggests that, poor social support during pregnancy could affect fetal growth and result in a low birth weight.

Majority of the participants had their anthropometric measurements within the normal ranges. A short, overweight, or obese woman had an increased odds of having a LBW baby. However, these asssocaitions were not statistically significant, and this is not surprising because one meta-analysis concluded that anthropometric measurements are not good predictors of LBW [28].

Iron deficiency has been reported as the commonest cause of anemia in pregnancy [29]. Anaemia in pregnancy is associated with an increased odds of having LBW babies [30], and our study findings supports association. A meta-analysis by Imdad has shown that iron supplementation significantly reduces the incidence of anemia in pregnancy by about 20%. Our study also revealed that mothers who never took daily required iron supplementation during pregnancy had a threefold increased odds of delivering a LBW baby [29].

Our study had a few limitations. We obtained mothers’ hemoglobin levels from their antenatal records and used it to assess anemia as a risk factor to LBW. Since mothers attend ANC clinic at different gestational ages. It would have been more accurate to follow the trends of their hemoglobin levels from the beginning of their antenatal period to delivery in assessing the association of anemia to LBW. Data on weight gain and height obtained from mothers’ antenatal record cards were the weights and heights of mothers at initiation of ANC, and therefore, associations between weight gain during pregnancy and actual BMI of the mother during pregnancy. These limitations notwithstanding, the study has revealed some important risk factors that may contribute to the occurrence of low birth weight in the Brong Ahafo Region.

## Conclusion

Preterm delivery, mothers with secondary education, living alone during pregnancy, not taking daily required iron supplementation and mothers with first trimester hemoglobin below 11 g/dl are determinants of LBW. Maternal anthropometric measurements do not influence birth weight. We recommend that the regional and the municipal health directorates in the Brong Ahafo region intensify education of all pregnant women on the need to take iron supplementation during pregnancy to prevent anemia and consequent LBW. Establishment of preconception clinics by the Ministry of Health to identify high risk pregnancies in all health facilities will add to the efforts at preventing negative birth outcomes.

## Abbreviations

ANC: Antenatal Care
BMI: Body Mass Index
CS: Caesarian section
DHIMS: District Health Information Management System
ELBW: Extremely Low Birth Weight
GDHS: Ghana Demographic and Health Survey
GSS: Ghana Statistical Service
ICD: International Classification of Disease
IUGR: Intrauterine Growth Restriction
LBW: Low birth weight
LMIC: Low and Middle Income Countries
MDG: Millennium Development Goals
PTB: Preterm birth
SGA: Small for Gestational Age
SVD: Spontaneous Vaginal Delivery
VLBW: Very Low Birth Weight
WHO: World Health Organization
